# P-38. Connecting the Dots: Unveiling Shared Motivations for Mpox Vaccination

**DOI:** 10.1093/ofid/ofae631.245

**Published:** 2025-01-29

**Authors:** Maurice Marshall, Shira Heisler, Paul Kim, Gretchen Newman, Lauren Touleyrou

**Affiliations:** Detroit Medical Center/Wayne State University, West Bloomfield, Michigan; DMC/Wayne State University, Detroit, Michigan; DMC/Wayne State University, Detroit, Michigan; Wayne State University School of Medicine, Rochester Hills, MI; Wayne State University, Detroit, Michigan

## Abstract

**Background:**

In 2022, a global outbreak of mpox spurred novel efforts for vaccination among previously unvaccinated individuals in the United States. Between 2022-2023, the United States was home to approximately 1/3 of documented global cases (32,063 cases) and suffered 56 deaths. These cases were concentrated in men-who-have-sex-with-men (MSM) and deaths were concentrated in people with HIV (PWH) and other immunocompromised individuals. Tolan Park Clinic in Detroit, Michigan, the largest HIV clinic in the state, began an intensive effort to vaccinate patients at risk of mpox acquisition. Surveys were provided to patients undergoing vaccination to assess motivations of undergoing vaccination. The objective of the study was to assess reasons for vaccination other than HIV status.

**Methods:**

Surveys were conducted of patients at Tolan Park Clinic who underwent mpox vaccination. During the survey period, 167 surveys were completed. The survey evaluated different motivations to receive vaccination, including exposure to mpox in the last 14 days, attendance at an event with a known case of mpox, MSM or transgender women with greater than two partners in the last year, MSM or transgender women with a partner known to have a STI in the last year, if pt participated in commercial sex work, or plan to attend a ball, sex party, or bath house. Patients could select multiple responses to the survey.

**Results:**

Analysis of results revealed that 49% (82) of surveys reported participants being MSM with greater than 2 partners in the last year as some motivation for undergoing vaccination. Following that, 24% (41) of surveys reported that participant planned to attend a ball, sex party, or bath house as a motivation for vaccination. The third most reported driver was MSM with a partner known to have an STI in the last year. This made of 14% (25) of all surveys reported.

**Conclusion:**

Results demonstrated that the two most common drivers of vaccination included MSM with greater than two partners in the last year and those planning to attend a ball, sex party or bath house. Targeting vaccination efforts toward these groups helped to curb the spread of mpox. Our data will be utilized to better target outreach efforts during future epidemics of mpox.

**Disclosures:**

**All Authors**: No reported disclosures

